# Combined Effects of Cold and Hot Air Drying on Physicochemical Properties of Semi-Dried *Takifugu obscurus* Fillets

**DOI:** 10.3390/foods12081649

**Published:** 2023-04-14

**Authors:** Ye Zhu, Xiaoting Chen, Kun Qiao, Bei Chen, Min Xu, Shuilin Cai, Wenzheng Shi, Zhiyu Liu

**Affiliations:** 1 Key Laboratory of Cultivation and High-Value Utilization of Marine Organisms in Fujian Province, National Research and Development Center for Marine Fish Processing (Xiamen), Fisheries Research Institute of Fujian, Xiamen 361013, China; yezhuzy@163.com (Y.Z.); xtchen@jmu.edu.cn (X.C.); qiaokun@xmu.edu.cn (K.Q.); chenbeifjfri@foxmail.com (B.C.); xumin@jmu.edu.cn (M.X.); caishuilin@hqu.edu.cn (S.C.); 2 College of Food Sciences & Technology, Shanghai Ocean University, Shanghai 201306, China; 3 College of Ocean Food and Biological Engineering, Jimei University, Xiamen 361021, China

**Keywords:** *Takifugu obscurus*, drying, physicochemical properties, microstructure, protein degradation

## Abstract

The physicochemical properties of semi-dried *Takifugu obscurus* fillets in cold air drying (CAD), hot air drying (HAD), and cold and hot air combined drying (CHACD) were analyzed based on pH, water state, lipid oxidation, protein degradation, and microstructure, using a texture analyzer, low-field nuclear magnetic resonance, thiobarbituric acid, frozen sections, sodium dodecyl sulfate polyacrylamide gel electrophoresis, and differential scanning calorimetry. Water binding to the samples was enhanced by all three drying methods, and the immobilized water content of CHACD was between that of HAD and CAD. The pH of the semi-dried fillets was improved by CHACD. When compared to HAD and CAD, CHACD improved the springiness and chewiness of the fillets, especially cold air drying for 90 min (CAD-90), with values of 0.97 and 59.79 g, respectively. The muscle fibers were arranged compactly and clearly in CAD-90, having higher muscle toughness. CHACD reduced the drying time and degree of lipid oxidation compared to HAD and CAD. CAD better preserved protein composition, whereas HAD and CHACD promoted actin production; CHACD had a higher protein denaturation temperature (74.08–74.57 °C). CHACD results in better physicochemical properties than HAD or CAD, including shortened drying time, reduced lipid oxidation, enhanced protein stability, and denser tissue structure. These results provide a theoretical basis for selecting the appropriate drying method for *T. obscurus* in industrial applications.

## 1. Introduction

Pufferfish (*Tetraodontidae*), belonging to *Tetraodontiformes*, is famous worldwide for its tetrodotoxin. Pufferfish abound in China with more than 40 species widely distributed in the East China Sea, Bohai Sea, Yellow Sea, and Yangtze River. *Takifugu obscurus* and *Takifugu rubripes* are the only two species of pufferfish that have been legally processed in China since 2016 [1]. Pufferfish meat is low in fat and high in protein, mineral elements, a variety of essential amino acids, and taurine, making it a fish with high nutritional value that is popular with consumers in Japan, Korea, and China [2]. According to the 2022 China Fishery Statistical Yearbook, the total amount of farmed pufferfish reached 29,950 tons in 2021, and the overall production will continue to increase [3]. However, the current processing capacity of pufferfish is higher than the supply, and pufferfish meat is not easily stored, as it only has a shelf life of about 4 d when refrigerated at 4 °C [4]. Therefore, extending the shelf life of pufferfish is important for improving production. In addition, the current processing method is too simple, as fish is mainly stored salt-dried and at low temperatures; this problem can be addressed by developing market-adapted pufferfish products.

Drying is an essential step in the aquatic products processing because it can extend shelf life, improve quality, and reduce storage and transportation costs by reducing the internal moisture content, thus inhibiting the growth of microorganisms and enzyme activity [5]. Traditional sun drying exhibits poor color, texture, and odor quality [6]. Hot air drying (HAD) in the processing of aquatic products—although higher temperatures can improve drying speed—involves the risk of destroying nutrients and physicochemical properties during the drying process [7]. Cold air drying (CAD) technology is appropriate for drying aquatic products with high protein content, as it can minimize the thermal denaturation of proteins, lipid oxidation, color change, and loss of flavor substances; however, the drying speed is slow [8]. Cold and hot air combined drying (CHACD) offers the advantages of both CAD and HAD, and has thus become a more popular method.

Different drying methods have different impacts on the water status, microstructure, lipid oxidation, and protein degradation of food products, leading to changes in their physicochemical properties [9]. Through drying, water evaporates within the aquatic product and the rate of lipid contact with oxygen increases [10]. During processing, moderate oxidation produces a satisfactory flavor, but over-oxidation leads to undesirable flavors [11], such as sourness and a disagreeable smell, and can even affect the health of consumers. The heating temperatures during drying can also lead to different degrees of denaturation of myofibrillar proteins, directly affecting their structure. Myofibrillar proteins are important functional proteins in meat products [12], and their denaturation results in coagulation and shrinkage, leading to significant changes in the texture of aquatic products, including springiness and hardness [13]. With increased drying time, more water evaporates from the surface of the material and muscle fibers contract, leading to the hardening of the tissue while hindering the outward diffusion of water. At the same time, the intermediates generated by lipid oxidation during drying cross-link with proteins through the Maillard and free radical reactions, causing protein denaturation and therefore a significant change in color after drying [14]. The selection of the appropriate drying method is necessary for maintaining the physicochemical properties of food products.

Hence, this study aimed to comprehensively analyze the physicochemical properties of semi-dried *T. obscurus* fillets by CAD, HAD, or CHACD. The effects of pH, water state, lipid oxidation, protein degradation, and microstructure were analyzed using texture analyzer, low-field nuclear magnetic resonance (LF-NMR), thiobarbituric acid (TBA), frozen sections, sodium dodecyl sulfate polyacrylamide gel electrophoresis (SDS-PAGE), and differential scanning calorimetry (DSC). The results obtained by these techniques provide a theoretical basis for selecting the most appropriate drying method for pufferfish fillets.

## 2. Materials and Methods

### 2.1. Sample Preparation

Two-year-old farmed *T. obscurus*, with a weight of 430 ± 30 g and a length of 26 ± 1 cm, was purchased from Fujian Tunzixian Aquatic Products Co., Ltd. (Zhangzhou, China). Living pufferfish were humanely slaughtered, gutted, skinned, and headed according to the guidelines issued by the Ministry of Agriculture of the P. R. of China (SC/T 3033–2016). The dorsal meat was washed with tap water and cut into pieces (20 × 15 × 5 mm, weight 2.0 ± 0.1 g) for drying. The pieces had an initial moisture content of 80 ± 1%.

### 2.2. Drying Conditions

The parameters of the different drying methods were optimized based on previous research [15], and the following methods were used to dry the fillets. HAD was performed using a hot air oven (BGZ-240, Boxun, Shanghai, China) for 37.5 min at 70 °C, with a wind speed of 1.5 m/s. CAD was operated using a cold air oven (HFD-2, Ouchen, Nanjing, China) for 146 min at 20 °C, with a relative humidity of 41% and a wind speed of 3 m/s. CHACD was performed using the above specified CAD and HAD parameters, with CAD performed for 30, 60, 90, and 120 min followed by HAD performed for 29, 28, 19, and 17 min. The final moisture content of all dried fillets was below 40 ± 1%.

### 2.3. Determination of pH

The pH was determined according to the method described by Fan et al. [16], with slight modifications. Approximately 2 g of fillets were dispersed in 18 mL of distilled water and homogenized using a T25 ULTRA-TURRAX^®^ (IKA Werke GmbH & Co., KG, Staufen, Germany). Using an Eppendorf 5810R high-speed freezing centrifuge (Eppendorf AG, Hamburg, Germany), the sample was centrifuged at 10,621× *g* for 10 min at 4 °C. The pH of the filtrate was measured using a digital pH meter (Mettler Toledo FE28, Shanghai, China) and measured three times in parallel.

### 2.4. Determination of Texture

The samples were investigated using an A/MORS P/5S probe (5 mm diameter, TA-XTplus, Stable Micro Systems, Godalming, UK), and a slight modification was made by referring to Guo et al. [17]. Two consecutive cycles at 50% sample deformation were applied at 1.5 mm/s test speed. Other parameters including pre-test speed, post-test speed and trigger force, were set at 2 mm/s, 2 mm/s, and 5 g, respectively. TPA analysis results were expressed in terms of hardness, chewiness, springiness, and resilience. Six parallel measurements were performed for each sample group.

### 2.5. LF-NMR Analysis

The protocol for LF-NMR analysis was slightly modified from that reported by Wang et al. [18]. A MesoMR LF-NMR analyzer (Shanghai Niumag Analytical Instrument, Shanghai, China) with a magnetic field strength of 0.5 T, corresponding to a spectrometer frequency (SF) of 21 MHz, was employed. The samples were transferred to the center of cylindrical glass tubes (25-mm radiofrequency coil) and equilibrated to 25 °C before detection. The transverse relaxation time (T_2_) was measured using a Carr–Purcell–Meiboom–Gill pulse sequence (CPMG). Data were acquired from 8000 echoes (NECH) over 16 scans (NS). The repetition time between scans was 3500 ms, and the 90 (P1) and 180 (P2) pulse times were 5 and 10 μs, respectively. The echo time (TE) was 0.2 ms, the number of sampling points (TD) was 320,026, the magnet frequency (SW) was 200 and regulate analog gain 1 (RG1) was 20 db. The MultiExp Inv analysis software Version 4.0 (Shanghai Niumag Analytical Instrument Co., Shanghai, China) based on the Simultaneous Iterative Reconstruction technique (SIRT) was used to obtain the single-component relaxation time (T_2W_) and transverse relaxation time (T_2_) distributions through mono-exponential or multi-exponential fitting. The samples were analyzed six times each.

### 2.6. Determination of Thiobarbituric Acid Value

The thiobarbituric acid (TBA) value was measured according to the method described by Wang et al. [19] with slight modifications. Three grams of minced fillet samples were homogenized in 30 mL of ice cooling 7.5% trichloroacetic acid solution containing 0.1% ethylene diamine tetraacetic acid (EDTA) at 15,000× *g* for 1 min. After filtering the homogenate, 5 mL of 0.02 M thiobarbituric acid was added to 5 mL of filtrate and mixed. The mixture was maintained in a boiling water bath for 40 min. After the solution had cooled, the absorbance of the upper layer was determined at 532 nm using a VICTOR Nivo^TM^ multimode plate reader (PerkinElmer Inc., Waltham, MA, USA). A standard curve was obtained using 1, 1, 3, 3-tetraethoxypropane and the results were expressed as mg of malonaldehyde (MDA)/kg. Each sample group was measured in triplicate.

### 2.7. Determination of Microstructure

The frozen sections were measured according to Sigurgisladottir et al. [20], with slight modifications. All samples were collected from the same location on each fillet, and the geometric centers of the fillets were cut into 5 × 5 × 5 mm pieces. The samples were embedded in plastic tubes containing O.C.T. compound (SAKURA Tissue Tek^®^, Torrance, CA, USA) and frozen in liquid nitrogen. Freezing occurred in approximately 40 s below −80 °C, and samples were then stored at −80 °C until sectioning. The specimens were sectioned (10 μm), frozen at −25 °C in a freezing microtome (Leica CM1950, Shanghai, China) for vertical cuts, and then placed on glass slides. After hematoxylin–eosin (HE) staining, the microstructures of the samples were examined using a fluorescent inverted microscope (DMi8, Leica Microsystems, Shanghai, China) using a 20× objective lens.

### 2.8. Protein Component Analysis

The method described by Yu et al. [21] was slightly modified. Briefly, semi-dried fillets of minced samples (5 g) were homogenized with 25 mL of Tris-HCI (20 mmol/L, pH 7.5) using a T25 ULTRA-TURRAX^®^ (IKA Werke GmbH & Co. KG, Staufen, Germany). The homogenate was centrifuged at 10,621× *g* for 10 min at 4 °C using an Eppendorf 5810R high-speed freezing centrifuge (Eppendorf AG, Hamburg, Germany). The precipitate was then re-homogenized and centrifuged again under the same conditions. The combined supernatants were treated with 10 mL of 50% trichloroacetic acid solution (TCA), left for 2 h, and then centrifuged at 10,621× *g* for 15 min. After centrifugation, the supernatant fractions were non-protein nitrogen (NPN) and precipitated as water-soluble protein (WSP). The precipitate obtained after the first centrifugation was added to 40 mL of 0.6 mol/L NaCl Tris-HCl buffer (20 mmol/L, pH 7.5), homogenized for 1 min, stirred for 1 h, and centrifuged at 10,621× *g* for 15 min at 4 °C. The supernatant was repeated twice for salt-soluble protein (SSP), and the residue was used as insoluble protein (ISP). The nitrogen contents of the different components were determined using the Kjeldahl method. Each group of samples was measured thrice in parallel. All the above operations were performed at 4 °C, and the solutions pre-cooled before use.

### 2.9. Sodium Dodecyl Sulfate Polyacrylamide Gel Electrophoresis Analysis

Extraction was performed following the method used by Setyabrata et al. [22], with a few modifications. Protein extraction was performed by homogenizing 0.1 g of fillet samples in 20 mL of whole protein extraction buffer (ratio of RIPA buffer (high) to PMSF was 100:1). The protein concentration of each sample was adjusted to 1 mg/mL using extraction buffer. Prior to sample loading, each sample was mixed well with its respective sample buffer. The samples were heated at 100 °C for 5 min in a loading buffer, cooled to 25 °C, and then 10 μL of sample solution was loaded into each well. The gels were prepared using 12% separating gel and 5% stacking gel. The gels were run on a Bio-Rad^®^ Criterion cell system (Bio-Rad^®^ Laboratories, Hercules, CA, USA) equipped with a DYY-6C electrophoresis apparatus (Beijing Liuyi Biotechnology Co., Ltd., Beijing, China) using 200 V constant voltage for 50–60 min. After running the gel, it was stained with 0.25% Coomassie brilliant blue R-250 (Bio-Rad, Richmond, CA, USA) and destained in a solution containing 5% ethanol and 10% acetic acid to visualize the gel. Gel visualization and image export were performed using an EPSON perfection V700 photo (Seiko Epson Co., Nagano, Japan). The protein bands were identified based on the determined molecular weight in parallel with the relative mobility of the protein bands to the dual-color pre-stained protein standard (10–250 kDa, EpiZyme, Shanghai, China).

### 2.10. Determination of Denaturation Temperature

Thermal transition properties were determined using differential scanning calorimetry (DSC; DSC 3, Mettler-Toledo International Trading Co., Ltd., Zurich, Switzerland). The method was performed according to Shang et al. [23]. After accurate weighing, 16–17 mg of each semi-dried sample was placed in a DSC aluminum pan, the added mass was recorded (accurate to 0.0001 g), and the pan was hermetically sealed. The initial temperature was set to 20 °C and the constant temperature time was 10 min. Then scanned from 20 to 100 °C, with a following temperature rise rate was of 5 °C/min at a nitrogen flow rate of 100 mL/min, and a reaction gas flow rate of 50 mL/min. A hermetically sealed empty pan was used as a reference. Three replicates were performed for each sample.

### 2.11. Statistical Analysis

One-way analysis of variance (ANOVA) followed by LSD and Duncan’s test were used to check for significant differences (*p* < 0.05) using SPSS Statistics 26 (SPSS Inc., Chicago, IL, USA). All experiments were performed three times, except for TBA and LF-NMR analyses, which were conducted in six replicates. Data are expressed as the mean ± standard deviation (SD) (*n* = 3). The data were plotted using OriginPro2018 (OriginLab Corp., Northampton, MA, USA). DSC data were analyzed using STARe Evaluation Software Version 16.20 (Mettler-Toledo International Trading Co., Ltd., Zurich, Switzerland) to create temperature plots.

## 3. Results and Discussion

### 3.1. Fillet pH

**Figure 1:**
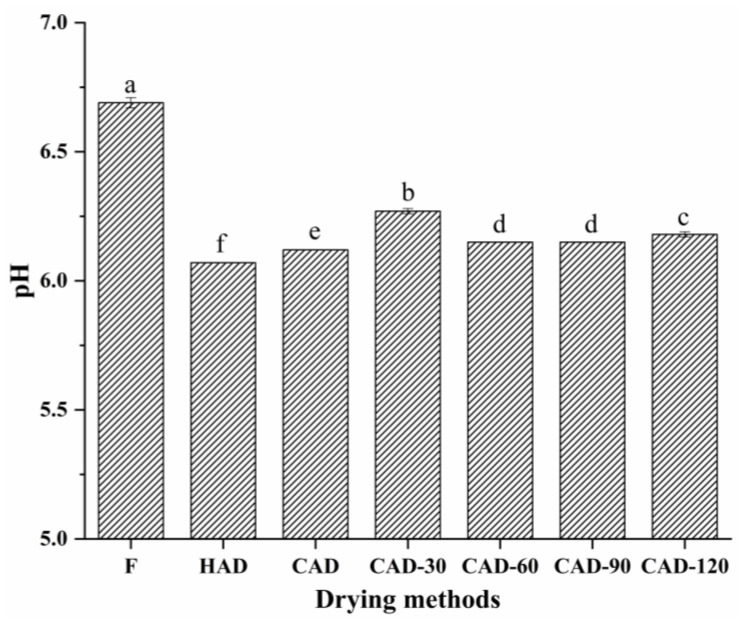
Changes in pH of semi-dried pufferfish fillets under different drying methods. Values with different superscripts are significantly different (*p* < 0.05). F, fresh undried fillets; HAD, hot air drying; CAD, cold air drying; CAD-30, cold air drying for 30 min; CAD-60, cold air drying for 60 min; CAD-90, cold air drying for 90 min; CAD-120, cold air drying for 120 min.

The pH is an important index for evaluating the quality of meat. Figure 1 shows the changes in the pH of pufferfish fillets under different drying methods. The pH of all fillets from all three treatments was weakly acidic, with that of fillets treated with HAD and CAD measuring 6.07 and 6.12, respectively, whereas that of CHACD-treated fillets was increased to 6.15–6.27. A strongly acidic environment leads to protein denaturation and decreases the water-holding capacity of muscle. Acidity is an important factor in flabby meat quality, and an increase in pH within a certain range results in improved meat quality [24]. However, increases in pH may be due to microbial production of nitrogenous substances and the production of substances, such as peptides and amines, by protein degradation during the drying process [25].

### 3.2. Fillet Texture

Texture is an important sensory attribute for consumer acceptance of aquatic products and influences the choice of subsequent processing methods [26]. Variations in pufferfish fillet textural properties, such as hardness, springiness, chewiness, and resilience under different drying methods, are shown in Table 1. CAD and CHACD helped maintain the hardness of the fillets, whereas HAD had the highest hardness (218.14 g) and chewiness (70.42 g). Hardening of tissue texture could be attributed to the rapid loss of surface water at higher temperatures and the appearance of hardened layers on the surface, along with increased protein denaturation and aggregation, contraction of myofibril and connective tissues, and reduction of extracellular space, intracellular lumens, and channels [27]. Similar results were reported by Vega-Gálvez et al. [28], where the hardness of giant squid meat increased dramatically after drying under different high-temperature conditions. Chewiness refers to the energy required to chew a solid to a swallowable state—the larger the value of elasticity, the better the taste of the fish. Appropriate increases in springiness and chewiness are beneficial for improving taste. Compared with HAD and CAD, CHACD improved springiness and chewiness, in which CAD-90 resulted in higher springiness and chewiness, with values of 0.97 and 59.79 g, respectively. The other three CHACD groups all had some correlation with CAD. This result may be due to the fact that CAD was performed first during CHACD, in that the connective tissue was less damaged, and the muscle fibers gradually contracted and tightly connected while water evaporated and dried to a certain extent before undergoing HAD, thus reducing the adverse effect of hardening of the sample surface during high-temperature drying. However, the resilience of all three drying methods was lower than that of the fresh samples, and there was no significant difference (*p* > 0.05). Changes in fillet texture after drying can be attributed to protein denaturation due to solute concentration, heat, and enzymatic denaturation [29].

### 3.3. LF-NMR of Fillets

The state and migration of water were further characterized using LF-NMR transverse relaxation time (T_2_). The relaxation time T_2_ is related to the degree of binding of the sample and hydrogen protons, and a higher binding of hydrogen protons indicates a lower mobility of water in the sample, which is expressed as a decrease in relaxation time T_2_ [30]. Bound water (T_20_ and T_21_) has a short relaxation time of 1–10 ms and represents water bound to macromolecules; immobilized water (T_22_) in the range of 10–100 ms indicates the water inside the myofibril and reticular tissues, which accounts for most of the total water content of the samples; and free water (T_23_) with the longest relaxation time mainly occurs between 100 and 1000 ms [31].

**Figure 2:**
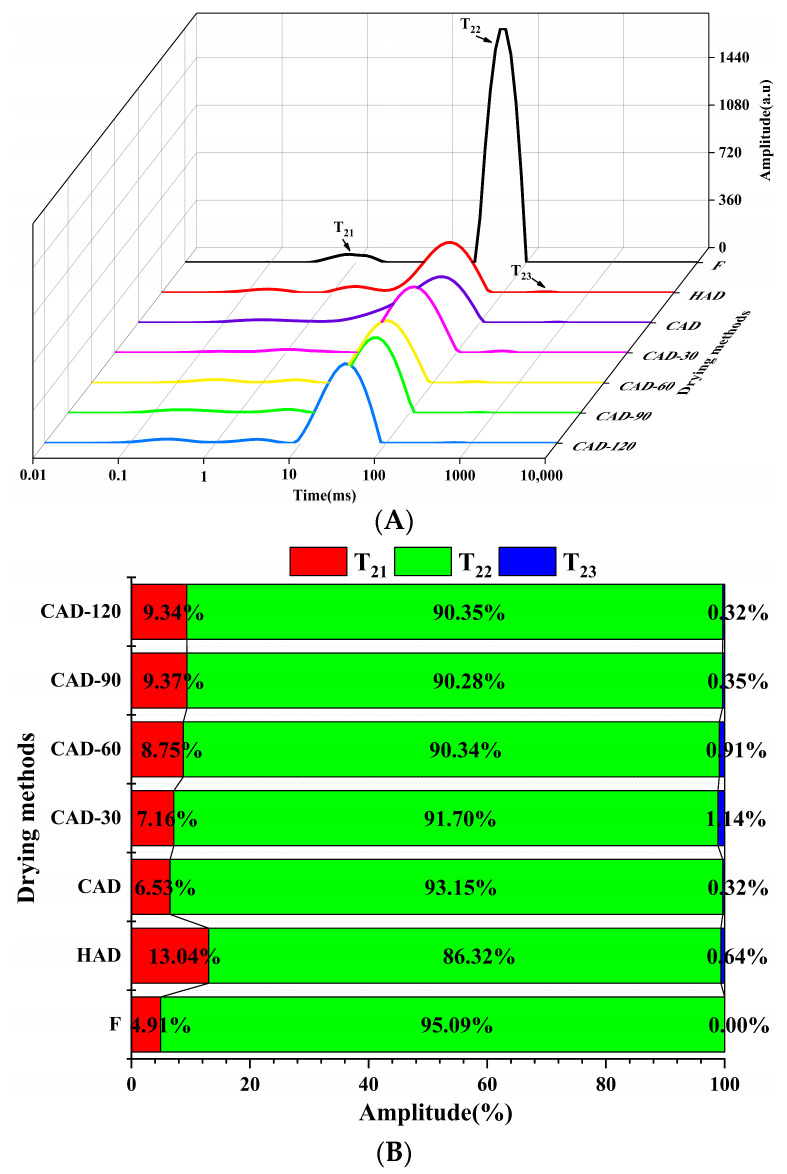
Curve of T_2_ transverse relaxation time (**A**) and proportion of various peak areas (**B**) in semi-dried pufferfish fillets. F, fresh undried fillets; HAD, hot air drying; CAD, cold air drying; CAD-30, cold air drying for 30 min; CAD-60, cold air drying for 60 min; CAD-90, cold air drying for 90 min; CAD-120, cold air drying for 120 min.

Figure 2A shows the relaxation time T_2_ curves of fresh pufferfish fillets obtained using the three drying methods, which resulted in a left shift of the peak positions of T_21_ and T_22_, a decrease in the peak area, and a shortening of the relaxation time T_2_. Across all three drying methods, the T_22_ of the fillets decreased when compared to T_21_ and T_23_, especially from 49.77 ms to 24.77 ms after HAD, indicating that the water binding to the sample increased and the mobility of immobilized water gradually decreased. This is consistent with the findings of Zhang et al. [32], in which the myofibril structure gradually shrank during drying, displaying a tighter network structure and enhanced water absorption capacity, resulting in a shorter T_2_ relaxation time. The relative percentages of water in the three states can be seen in Figure 2B, and the content of immobilized water in the undried fresh samples was higher, accounting for 95.09% of the total water content; the fillets mainly contained bound and immobilized water, and the content of immobilized water was reduced in all four groups of CHACD, and between HAD and CAD. The increase in the T_21_ peak area was mainly due to the conversion of immobilized water into free water and its subsequent outward diffusion during the drying process. The T_23_ peak area increased in all drying methods, with the highest after CAD-30 containing 1.14%, which may be due to the destruction of the protein structure and the release of immobilized water from the outer parts of myofibrils [33].

### 3.4. Oxidation of Fillet Lipids

**Figure 3:**
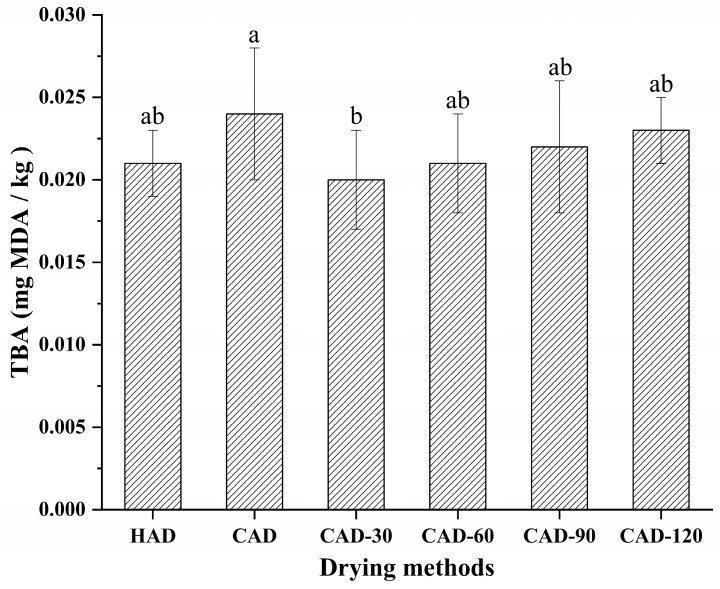
Changes in thiobarbituric acid (TBA) values in semi-dried pufferfish fillets. Values with different superscripts are significantly different (*p* < 0.05). HAD, hot air drying; CAD, cold air drying; CAD-30, cold air drying for 30 min; CAD-60, cold air drying for 60 min; CAD-90, cold air drying for 90 min; CAD-120, cold air drying for 120 min.

The TBA value corresponds with the content of malondialdehyde (MDA) produced during lipid oxidation, which is a natural and final product of lipid peroxidation [34]; therefore, the degree of lipid oxidation in aquatic products can be expressed as TBA [14]. Figure 3 shows the variation in TBA values of semi-dried pufferfish fillets treated with different drying methods. The three drying methods had some effect on the lipid oxidation of pufferfish fillets. CAD had the largest TBA value of 0.024 mg MDA/kg among the three drying methods, indicating that the effect of CAD on the lipid oxidation of fillets was more obvious, which may be due to the relatively longer exposure of fish fillets to air [35] and increased oxidation of unsaturated fatty acids during dehydration [36]. Compared with CAD, CHACD reduced the degree of lipid oxidation by reducing the drying time. The degree of lipid oxidation in CHACD was similar to that in HAD (*p* < 0.05).

### 3.5. Fillet Microstructure

**Figure 4:**
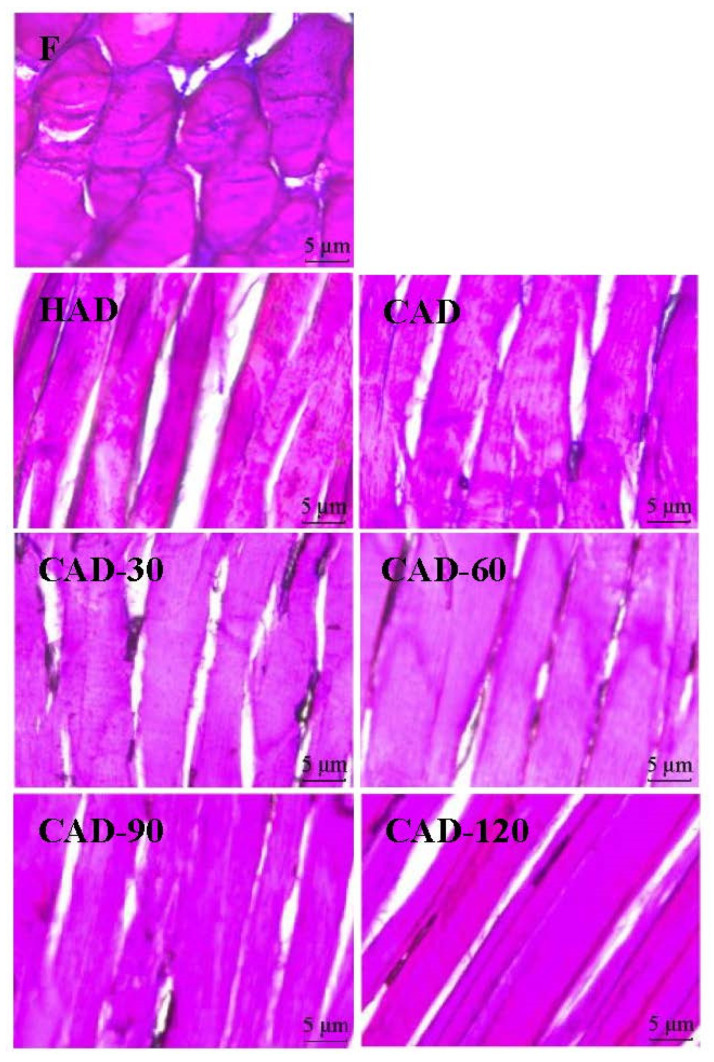
Microstructure (vertical section) of semi-dried pufferfish fillets under different drying methods. F, fresh undried fillets; HAD, hot air drying; CAD, cold air drying; CAD-30, cold air drying for 30 min; CAD-60, cold air drying for 60 min; CAD-90, cold air drying for 90 min; CAD-120, cold air drying for 120 min.

The changes in fillet microstructure (vertical section) under different drying methods are shown in Figure 4. The microstructure is closely related to the quality of the fillets after drying and can indicate the degree of protein degradation [37]. As shown in Figure 4, the muscle fibers of the undried fresh fish samples were intact and well defined. Using different drying methods, the muscle fibers showed different degrees of shrinkage. After HAD, the muscle fibers were firmer but showed fiber breakage and significant degradation. This may be due to the higher temperature, which accelerates protein denaturation, and the smaller size of myofibrils and collagen, resulting in a reduction in muscle fiber diameter and length [27]. Fillet microstructure after CAD had smaller and well-defined muscle fiber gaps, which may be due to the lower temperature and longer drying duration, which allows sufficient time for muscle fiber contraction and reduced protease activity, thus mitigating protein degradation [38]. In contrast, CHACD-treated fillets more and more resembled the microstructure of CAD-treated fillets with increasing CAD treatment time, and the muscle fibers changed from partially broken to intact, well-arranged, and well-defined. Among them, the muscle fiber structure of CAD-90-treated fillets was the most arranged, compact, well-defined, and complete in structure among the four CHACD groups, showing high muscle toughness. This indicated that CAD-90 resulted in better texture properties, which was consistent with the results of the texture analysis.

### 3.6. Fillet Protein Composition

According to the difference in fish protein solubility, pufferfish proteins were divided into WSP, SSP, ISP, and NPN. The changes in the protein composition of pufferfish fillets subjected to different drying methods are summarized in Table 2. The WSP content in fresh fillets was the highest, accounting for 58.09% of the total protein, followed by ISP, accounting for 26.76%, whereas the content of SSP and NPN was lower. The NPN content increased but varied less among the three drying methods. NPN is composed of free amino acids, small peptides, and nucleic acids [39]. With the three drying methods, the moisture content in the fillets was reduced, which helped to inhibit microbial and enzymatic activities. However, drying degraded proteins with a large molecular mass to produce free amino acids and peptides, thereby increasing the NPN content. When compared to the fresh samples, the WSP content of the two drying methods decreased and was not significantly different (*p* > 0.05), except for CAD, which could better maintain WSP. When comparing the four CHACD groups, the WSP content tended to increase slightly with an increase in CAD time. This may be due to a decrease in WSP solubility as a result of protein concentration. For CAD, this may be due to a decrease in WSP solubility as a result of protein concentration due to altered water loss at low temperatures. For HAD, this may be due to WSP denaturation caused by high temperature, which leads to a decrease in WSP solubility. However, CHACD combines the characteristics of both drying methods, leading to a decrease in solubility [40]. When compared to the undried fresh samples, the SSP of CAD significantly increased from 6.93 to 8.15 mgN/g. However, the SSP content of HAD and CHACD significantly decreased (*p* < 0.05), with the four CHACD groups increasing with increasing CAD time. When comparing the three drying methods, ISP increased significantly (*p* < 0.05), and the content in CHACD decreased with increasing CAD time, with the largest increases in CAD-30 and CAD-60, with 19.32 and 18.69 mgN/g, respectively. The significant increase in ISP in HAD and CHACD may be related to the aggregation of WSP and SSP by thermal denaturation and the fact that protein fractions in fish fillets are more unstable during HAD, leading to a significant increase in ISP content [21].

Proteins play a key role in determining the physical properties of meat products. Han et al. [41] also found that heat induction caused a negative correlation between the degree of pork myofibrillar protein aggregation and immobilized water content and showed that protein aggregation tends to form network structures. This is consistent with the LF-NMR and microstructure analysis, where the relative percentage of the immobilized water content was lower in HAD than in CAD and CHACD, and the myofibrils were more tightly arranged in the HAD and CHACD groups.

### 3.7. Degradation of Fillet Proteins

**Figure 5:**
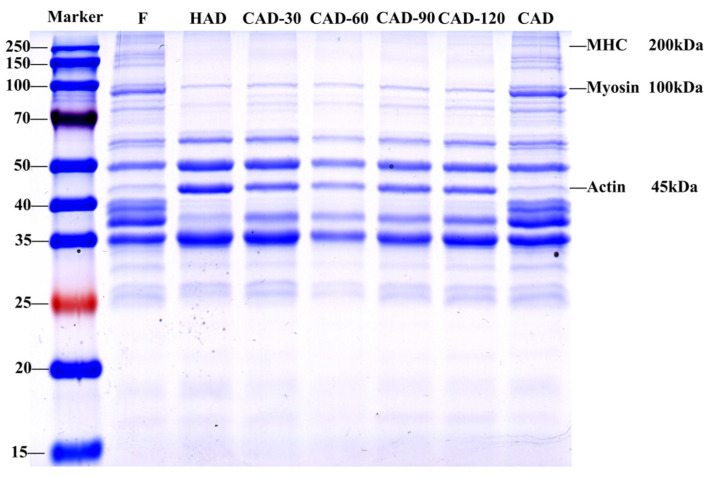
SDS-PAGE of myofibrillar proteins from semi-dried pufferfish fillets dried using different methods. F, fresh, undried fillets; HAD, hot air drying; CAD, cold air drying; CAD-30, cold air drying for 30 min; CAD-60, cold air drying for 60 min; CAD-90, cold air drying for 90 min; CAD-120, cold air drying for 120 min.

SDS-PAGE can be used to determine molecular weight and to analyze the number of subunits in protein molecules [42]. We analyzed three common proteins: myosin heavy chain (MHC, 200 kDa), myosin (100 kDa), and actin (45 kDa) [43]. Figure 5 shows the changes in SDS-PAGE of fillets treated using different drying methods. The MHC and myosin bands were clearly visible after CAD when compared to fresh samples, and the actin band remained unaltered. In HAD and CHACD, the MHC band disappeared and myosin remained unaltered, whereas the actin band became thicker and the color deepened to be more clearly visible. CHACD showed an increase, then a decrease, and then an increase in the actin band with the increase in CAD time, indicating that heating could reduce the thermal stability of myosin and actin and accelerate protein degradation. Zheng et al. [44] also found that actin bands disappeared more slowly than myosin bands during heating of chicken meat myofibrillar proteins.

### 3.8. Thermal Stability of Fillet Proteins

**Figure 6:**
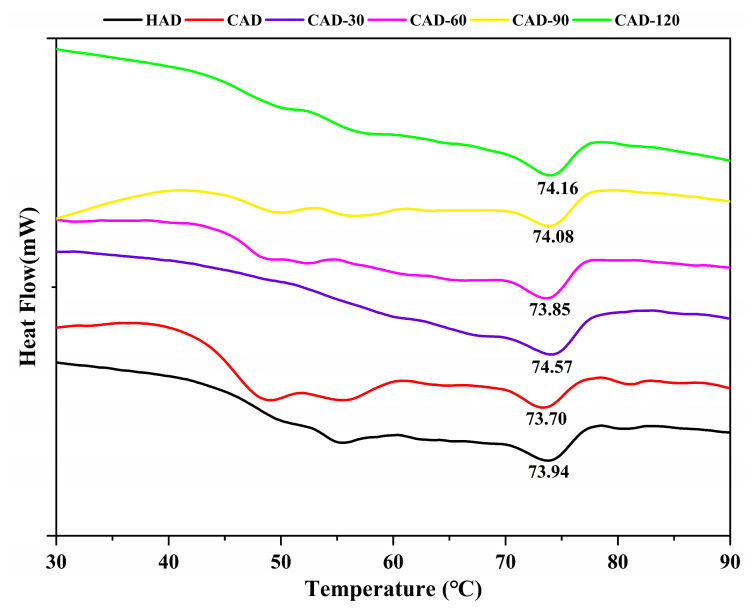
Differential scanning calorimetry of semi-dried pufferfish fillets under different drying methods. F, fresh, undried fillets; HAD, hot air drying; CAD, cold air drying; CAD-30, cold air drying for 30 min; CAD-60, cold air drying for 60 min; CAD-90, cold air drying for 90 min; CAD-120, cold air drying for 120 min.

Thermal stability is an important measure of protein stability in aquatic products. In general, the treated fish had two heat absorption peaks near 50 and 75 °C for myosin (T_P1_) and actin (T_P2_) [45], respectively, where myosin is the most unstable of the structural proteins and completes the entire denaturation process between 40–60 °C [46]; actin is the most thermally stable protein, starting to denature at 71 °C, endoscopically transitioning between 78 °C, and denaturing completely when heated to approximately 83 °C [23]. Figure 6 shows the two peak values of endothermic transitions in samples treated with different drying methods. The T_P1_ value after HAD (52.50 °C) was higher than that after CAD, whereas all the CHACD myosins underwent different degrees of thermal denaturation. While the T_P2_ after HAD (73.94 °C) was higher than that after CAD (73.70 °C), it was lower than that after all CHACD treatments (74.08–74.57 °C), except for CAD-60 (73.85 °C), indicating that CHACD could also effectively reduce the degradation of actin, which was consistent with the results of SDS-PAGE. The enthalpy of denaturation (ΔH) of the samples treated with the three drying methods varied from 0.11–0.42 (peak I) and 0.21–0.29 (peak II), indicating that the increase in heating temperature was associated with a decrease in Δ [47]. 

## 4. Conclusions

We compared the effects of CAD, HAD, and CHACD on the physicochemical properties of *T. obscurus*, including the pH, water state, lipid oxidation, protein degradation, and microstructure. All three drying methods enhanced the binding of water to the sample, existing mainly in a bound or immobilized form that was converted into free water to diffuse outward. The content of CHACD-immobilized water was between that of HAD and CAD. The pH after HAD and CAD was low, whereas CHACD increased the pH to some extent, but the fish treated with all three drying methods were weakly acidic. HAD-treated fillets had the highest hardness and chewiness, but fiber breakage and degradation were observed. When compared to HAD and CAD, CHACD improved the springiness and chewiness of the fillets, among which CAD-90 resulted in higher springiness and chewiness and showed higher muscle toughness among the four CHACD groups. All three drying methods resulted in some degree of lipid oxidation. The TBA value of CAD was the highest among the three drying methods, whereas CHACD reduced the drying time and decreased the degree of lipid oxidation through combined drying. In terms of protein degradation, CAD better maintained the protein composition, while HAD and CHACD promoted the formation of actin, and CHACD had a higher protein denaturation temperature. We found that CHACD combines the advantages of CAD and HAD with respect to effects on the physicochemical characteristics of semi-dried pufferfish fillets, including shortened drying time, reduced degree of lipid oxidation, enhanced protein stability, and denser tissue structure. This study provides a theoretical basis to improve research on and development of the most suitable drying method for semi-dried *T. obscurus* in industrial applications.

## Figures and Tables

**Table 1:** Texture characteristics of pufferfish fillets under different drying methods.

Drying Methods	Hardness (g)	Springiness	Chewiness (g)	Resilience
F	133.60 ± 23.55 ^b^	0.91 ± 0.02 ^ab^	53.57 ± 8.05 ^b^	0.28 ± 0.03 ^a^
HAD	218.14 ± 28.28 ^a^	0.60 ± 0.04 ^c^	70.42 ± 11.95 ^a^	0.16 ± 0.01 ^c^
CAD	93.96 ± 10.35 ^b^	0.88 ± 0.07 ^b^	56.62 ± 6.74 ^ab^	0.20 ± 0.03 ^b^
CAD-30	101.04 ± 22.89 ^b^	0.87 ± 0.06 ^b^	55.68 ± 10.53 ^ab^	0.21 ± 0.02 ^b^
CAD-60	105.54 ± 23.72 ^b^	0.88 ± 0.05 ^b^	59.57 ± 14.48 ^ab^	0.19 ± 0.01 ^b^
CAD-90	102.27 ± 11.70 ^b^	0.97 ± 0.02 ^a^	59.79 ± 6.26 ^ab^	0.19 ± 0.02 ^b^
CAD-120	90.48 ± 19.07 ^b^	0.88 ± 0.10 ^b^	49.88 ± 14.53 ^b^	0.18 ± 0.01 ^bc^

Data are expressed as the mean ± SD (*n* = 6). Values within a column with different superscripts are significantly different (*p* < 0.05). F, fresh undried fillets; HAD, hot air drying; CAD, cold air drying; CAD-30, cold air drying for 30 min; CAD-60, cold air drying for 60 min; CAD-90, cold air drying for 90 min; CAD-120, cold air drying for 120 min.

**Table 2:** Protein composition of pufferfish fillets under different drying methods (mgN/g).

Drying Methods	NPN	WSP	SSP	ISP
F	0.20 ± 0.01 ^c^	27.33 ± 0.71 ^a^	6.93 ± 0.40 ^b^	12.59 ± 0.86 ^c^
HAD	0.26 ± 0.01 ^a^	19.37 ± 0.43 ^b^	3.56 ± 0.32 ^e^	15.57 ± 1.50 ^b^
CAD	0.23 ± 0.01 ^b^	26.80 ± 1.73 ^a^	8.15 ± 0.14 ^a^	15.53 ± 0.69 ^b^
CAD-30	0.27 ± 0.01 ^a^	18.63 ± 0.32 ^b^	3.55 ± 0.04 ^e^	19.32 ± 0.94 ^a^
CAD-60	0.27 ± 0.01 ^a^	19.60 ± 1.77 ^b^	3.78 ± 0.11 ^de^	18.69 ± 0.98 ^a^
CAD-90	0.27 ± 0.01 ^a^	20.30 ± 1.46 ^b^	4.33 ± 0.33 ^d^	17.60 ± 0.53 ^ab^
CAD-120	0.26 ± 0.01 ^a^	20.51 ± 0.39 ^b^	5.39 ± 0.35 ^c^	15.93 ± 0.39 ^b^

Data are expressed as the mean ± SD (*n* = 6). Values within a column with different superscripts are significantly different (*p* < 0.05). NPN, non-protein nitrogen; WSP, water-soluble protein; SSP, salt-soluble protein; ISP, insoluble protein; F, fresh undried fillets; HAD, hot air drying; CAD, cold air drying; CAD-30, cold air drying for 30 min; CAD-60, cold air drying for 60 min; CAD-90, cold air drying for 90 min; CAD-120, cold air drying for 120 min.

## Data Availability

Data is contained within the article.
